# The Role of T_RM_ Cells in the Pathogenesis of Vitiligo—A Review of the Current State-Of-The-Art

**DOI:** 10.3390/ijms21103552

**Published:** 2020-05-18

**Authors:** Alicja Frączek, Agnieszka Owczarczyk-Saczonek, Waldemar Placek

**Affiliations:** School of Medicine, Collegium Medicum, The University of Warmia and Mazury, 10-229 Olsztyn, Poland; aganek@wp.pl (A.O.-S.); waldemar.placek@uwm.edu.pl (W.P.)

**Keywords:** vitiligo, tissue-resident memory T cells, IL-15, autoimmune diseases

## Abstract

Vitiligo is the most common hypopigmentation disease affecting both the skin and mucous membranes. The pathogenesis of this disorder is complex and involves the influence of genetic and environmental factors, oxidative stress, and autoimmune responses. Recent studies have indicated that skin lesions observed in vitiligo tend to recur in the same places where they were found before treatment. This phenomenon is explained by the presence of recently discovered tissue-resident memory T cells (T_RM_), whose primary function is to provide antiviral and antibacterial protection in non-lymphoid tissues. T_RM_ cells show the presence of CD49a, CD69, and CD103 markers on their surface, although not all of them express these particles. Due to their ability to produce and secrete perforin, IFN-γ, and granzyme B, T_RM_ cells demonstrate a cytotoxic effect on melanocytes, thus inducing depigmented lesions in the course of the vitiligo. It has been proved that the occurrence of T_RM_ cells largely depends on IL-15, which promotes the T_RM_ function ex vivo. The findings above, as well as their reference to the pathogenesis of autoimmune skin diseases will have a considerable influence on the development of new therapeutic strategies in the near future. This article presents an up-to-date review of information regarding the role of T_RM_ cells in the development and progression of vitiligo.

## 1. Introduction

Vitiligo is a disease characterized by white patches of different shapes and sizes, which appear on the skin, overlying hair, and mucous membranes. Depigmentation is a result of the destruction of melanin-producing cells, called melanocytes, which embryologically origin from neutral crest and are located in the hair bulbs and basal layer of epidermis [1]. Interestingly, the word “vitiligo”, is derived from the Latin “vitium”, meaning “blemishing fault” [2]. The lesions often appear on elbows, knees, wrists, or the back of hands, i.e., in places most exposed to sunlight. They also show up around natural body orifices, such as the mouth, nose, eyes, and genitourinary organs [3,4]. Although vitiligo was described thousands of years ago (some records come from ancient Egypt and India), there is still a negative social stigma connected with this disease [5]. Consequently, pigmentary disfigurement might be a considerable cosmetic problem, seriously affecting the quality of patients’ lives by decreasing their sense of self-esteem and causing negative feelings, such as depression or anxiety [6]. Importantly, vitiligo is fully reversible when appropriate treatment is applied. Nevertheless, current therapies help to achieve only short-term benefits [5]. In the past, various methods were used, which were supposed to stop the disease. The repigmentation procedure was based on cow dung, cobra snake bones, or toxic substances, like arsenic [5]. Today, there are many options available, including topical corticosteroids and calcineurin inhibitors, PUVA light therapy, narrow-band UVB (nbUVB) phototherapy, or even surgery. It depends on the patient’s age, overall health, and preferences. However, the most successful treatment effects are based both on immunosuppression and simultaneous regeneration of melanocytes [5,7].

Apart from the skin, melanocytes also occur in the uveal layer of the eye, vaginal epithelium, and endolymphatic sac of the inner ear [1,8]. In spite of the fact that the disease does not usually influence melanocytes residing in these tissues, some studies indicate the connection between loss of hearing and vitiligo, regardless of the duration of the disease [1]. Besides the peripheral auditory system, melanocytes are also present in the central auditory system [1,9]. Even though the functions of otic melanocytes are not fully recognized, it seems that they play a protective role in the hearing process [10]. Hospital-based studies conducted by Mahdi et al. demonstrated that sensory neural hearing loss was present in approximately 40% of patients with diagnosed vitiligo [1]. This result suggests that the melanocytotoxic effect is not only a cutaneous problem. Because of this, some researchers recommend that vitiligo diagnosis should also include pure tone audiometry and ABR measurement, irrespective of any hearing problem. In addition, patients should avoid ototoxic drugs or any other noise exposure, particularly during disease activation [11]. MITF (microphthalmia-associated transcription factor) is a protein encoded by *Mitf* gene in humans, which induces melanin synthesis by regulating the expression of melanogenic enzymes, including tyrosinase-related protein-1 (TRP-1) and 2 (TRP-2). Mutations in this gene are detected in the diseases with pigmentation and deafness background, therefore it can be supposed that this factor, apart from the influence on the melanocytes, is also required in the hearing process [12,13].

The vitiligo prevalence ranges from 0.1% to 2% in most populations around the world, with no significant differences between the sexes [3]. The occurrence varies between geographic regions [14], but globally, vitiligo remains the most common hypopigmentation disorder [15]. The latest research, focusing on the ethiopathogenesis of this disease, shows that apart from genetic and environmental factors, the development of vitiligo is strongly related to autoimmune processes [16]. This theory is supported by a recent discovery of a new population of memory T cells, called T_RM_, which, by initiating the inflammatory process, are able to cause recurrent vitiligo lesions in the same places where an effective therapy has been applied before. There are still several concepts regarding the primary role of T_RM_ cells in immune response. In some studies, skin T_RM_ cells characterized by promoting local inflammation do not recruit other effector T cells from blood circulation. In another concept, the major function of T_RM_ cells is the production and secretion of cytokines which are responsible for T cells recruitment [5]. The study conducted by Frisoli et al. demonstrated that selective depletion of recirculating memory T cells or inhibition of their migration contributed to rapid repigmentation, in spite of the fact that the number of T_RM_ cells did not change [5]. This observation led to the conclusion that T_RM_ cells are not fully responsible for relapsing skin lesions in vitiligo without additional recruiting of T cells [5]. This seems to be important information due to the fact that ~40% of patients suffer from a disease episode within the first year after finishing treatment [17]. By secreting compounds like granzyme B, perforin, or IFN-γ, T_RM_ cells exert a cytotoxic effect on melanocytes, leading to their apoptosis. Importantly, CD8^+^ T_RM_ cells which are present in healthy human skin do not demonstrate high expression of these effector molecules [5]. Apart from the skin, this long-living subset of T cells is also found in many other peripheral tissues including brain, liver, kidneys, spleen and heart. In the mucosa, T_RM_ cells have been detected in the gut, female reproductive tract and lungs. They are also present in secondary lymphoid organs, which represent the first line of defense against infection factors [18,19].

## 2. Vitiligo Classification

In 2011, during the International Pigment Cell Conference (IPCC), two main forms of vitiligo were distinguished: segmental (SV) and non-segmental (NSV). It is noteworthy that the term “vitiligo” refers to all non-segmental forms, including the following subtypes: acrofacial, mucosal (more than one mucosal site), universal, generalized, mixed and other rare variants (Table 1) [20].

Generally, NSV refers to depigmented skin lesions that demonstrate a tendency to symmetrical localization, while SV refers to uni-, bi-, or plurisegmental white patches with earlier age of onset than NSV [20]. Acrofacial vitiligo is mainly located on the face and/or distal parts of the body, especially fingers. We can distinguish a subcategory of the acrofacial type, the lip-tip variety, which refers to the cutaneous lips and the distal tips of the fingers. Sometimes, acrofacial vitiligo demonstrates an ability to progress to a generalized or universal form of disease [20,21]. Generalized vitiligo usually refers to a symmetrical, bilateral depigmented skin area, which occurs over the entire body [21]. Usually, mucosal vitiligo is present on oral and/or genital mucosae. An isolated form is classified as undetermined vitiligo, while mucosal vitiligo, in the context of generalized vitiligo, is classified as NSV [20,21]. The term “universal vitiligo” is used to describe skin lesions that cover approximately 80–90% of body surface. This form of hypopigmentation disorder is a common consequence of generalized vitiligo progression with melanocyte destruction, not only from the skin but also the body hair and, rarely, oral/genital mucosae [20,21]. Focal vitiligo is characterized by small isolated white patches, without typical segmental distribution. This subtype can be diagnosed if after 1–2 years there is no progression to NSV [20]. Mixed vitiligo displays clinical features of SV and NSV [22]. This subtype was first noticed in a pediatric patient with NSV, treated with the UVB narrow band therapy, which revealed a segmental depigmented skin area on the child’s body. The difference in response to the treatment suggested a coexistence of SV and NSV [23]. The rare forms of vitiligo include vitiligo punctate (if depigmented macules do not occur with classical vitiligo skin lesions, than the term ‘leukoderma punctata’ is used), hypochromic vitiligo (also referred to as ‘vitiligo minor’) and follicular vitiligo [20,21]. SV is usually characterized by notable progression with hair follicle melanocytes destruction called poliosis [20]. In contrast to SV, NSV generally does not affect hair bulbs, but due to vitiligo progression, a decrease or absence of melanin may take place [20]. Because of clinical differences between SV and NSV, in the past, it was supposed that these two forms of skin disorder are connected with distinct pathogenetic processes. Nevertheless, further studies have pointed to the contribution of inflammatory and immunity-related mechanisms for both SV and NSV [21,24]. Distinguishing these two major types of vitiligo is very important from the prognostic point of view [21]. In spite of the fact that the final presumptive area of the segment involved is one of the most important issues for a patient with SV, there is still no international agreement on this question due to the scarcity of data [20]. The Vitiligo European Task Force (VETF) is a grading system that scores the extent, stage, and progression of disease based on skin and hair pigmentation connected with melanocyte reservoir. It seems to be a suitable prognostic option for NSV, so it should be tested in clinical trials [20,25].

## 3. The Pathogenesis of Vitiligo

Vitiligo is probably the result of an interaction between genetic, environmental, and biochemical factors supporting autoimmune processes, in which melanocytes are destroyed on the body areas affected by the disease. Considering the high incidence (11–31%) of developing this skin disorder in the family, as well as the early age when the first symptoms are observed (usually in patients aged 10–30; over half of the cases are patients below 20), it has been suggested that genetic predispositions contribute to the development of vitiligo [4,26,27,28]. In addition, a study conducted by Alkhateeb et al. demonstrated that the outbreak age of the disease is lower in family cases [3]. This concept is supported by the fact that, compared to general population, vitiligo occurs more frequently (18 times) among siblings [3]. Genome analysis of multigeneration families with generalized depigmentation lesions and other associated diseases indicated that vitiligo is an autosomal dominant pattern with incomplete penetrance [29]. Other studies, based on familial aggregation, suggested that polygenic, multifactorial inheritance explains partial heritability [15]. It is interesting that the concordance for vitiligo in monozygotic twins was 23%, demonstrating a 60-fold increased risk in comparison with general population [3,15]. Additionally, these results suggest that apart from genetic factors, other components are also important for the development of this depigmenting skin disorder [3,15]. Further observations confirmed that the major genetic risk of vitiligo is strongly connected with polymorphism in HLA-A, HLA-DRB1/DQA1, and CPVL [5]. In fact, HLA genes are responsible for antigen presentation, while CPVL is postulated to play a role in antigen processing [30,31].

By means of the Genome Wide Association Study (GWAS), it was proved that vitiligo patients, as well as their relatives, are more exposed to other autoimmune diseases. Several genes have been identified which seem to be associated with both generalized vitiligo and other autoimmune/autoinflammatory diseases [15,30]. Usually, these patients show higher values of antibodies to thyroid peroxidase and thyroglobulin compared to the general population. Moreover, we can observe a greater incidence of rheumatoid arthritis, psoriasis, type 1 diabetes mellitus, pernicious anemia, systemic lupus erythematosus or Addison’s disease [5,28,29]. The GWA study indicated nearly 50 *loci* associated with genes controlling the innate (NLRP1, IFIH1, casp7, c1qtnf6, trif) and acquired (FOXP3, BACH2, CD80, CCR6, PTPN22, IL2R, αG2MB, HLA class I and II) immunity system [15,32,33,34]. This finding may, to a certain extent, explain recruitment of cells such as NK (natural killer), as well as increased production and secretion of proinflammatory proteins and cytokines including IL-1β, IL-6, IL-8 and heat shock proteins (HSP), especially HSP70i, participating in vitiligo progression [32,34,35]. Recently, it has been found that NLRP1, which is a gene located on chromosome 17p, is responsible for the regulation of the innate immune system, stimulation of IL-1β secretion and apoptotic process by coding an apoptosis protein from Ced-family. In addition, NLRP1 is a susceptibility gene for generalized vitiligo and other autoimmune vitiligo-associated diseases [33,36].

The depigmentation process is connected with both the decreasing number of melanocytes and their slower proliferation in comparison with healthy skin [37]. Despite the fact that the mechanisms of vitiligo pathogenesis are described in many studies, it is unclear which factor is crucial for activating melanocytes destruction. Some hypotheses suggest dysregulation in redox balance, which is strongly connected with lower expression of catalase, an enzyme which has a major role in protection from oxidative damage caused by reactive oxygen species (ROS) [5,38]. Cellular stress can directly influence melanocytes, stimulating them to release DAMPs (damage-associated molecular patterns). As a consequence, a noninfectious inflammatory reaction with recruitment of dendric cells (DCs), NK cells and CD8^+^ T cells to vitiligo lesions is initiated [39]. Chemical compounds such as 4-TBP (4-tertiary butyl phenol) or MBEH (monobenzyl ether of hydroquinone) are known to partake in ROS induction and activation of the unfolded protein response (UPR). The transcription factor x-box-binding protein 1 (XBP1) is a key UPR component demonstrating an increase due to an exposure to chemical triggers. It is partly responsible for regulating the production and secretion of proinflammatory cytokines IL-6 and IL-8 by melanocytes. Both of these interleukins are believed to influence the attraction of T-cells to depigmented skin lesions [40]. Furthermore, IL-6 has an impact on the expression of Intercellular Adhesion Molecule 1 (ICAM-1) on melanocytes, which might induce cytotoxic response by stimulating leukocyte-melanocyte attachment [41]. Interestingly, some studies point to the connection between the higher level of IL-6 in the tissue and/or serum and the presence of autoimmune disorders accompanying vitiligo [42,43]. Additionally, as a result of the oxidative stress, keratinocytes show growing expression of chemokine CXCL16, which has an influence on the migration of lymphocytes T CD8^+^ CXCR6^+^. The infiltration of these cells leads to decreasing the number of melanocytes within the area of skin lesions in patients with vitiligo [44].

CD4^+^ helper T cells are mainly responsible for activation of other immune cells by producing cytokines in response to antigen stimulation. Some studies suggest that in lymphocytes Th CD4^+^, Th1 and Th17 are the major subset in vitiligo development [45]. Patients with melanocytes destruction display a heightened level of cells Th17 [46]. It has been proved that this subset of helper T cells is involved in expression of IL-17A, IL-17F, as well as IL-22 [47,48]. In turn, IL-22 affects keratinocytes by stimulating them to secretion of IL-1β, also known as a mononuclear cell factor, inducing in this way an inflammatory response. In addition, IL-β, next to IL-1 and tumor necrosis factor (TNF-α), is a significant cytokine which demonstrates an influence on Th17 cell development [48]. Moreover, Th17 cells demonstrate ability to secrete IL-6 and also stimulate keratinocytes to express this interleukin [49]. Finally, a higher expression of IL-17 was noticed in the lesional edge, which seems to explain a possible role of lymphocyte Th17 in the pathogenesis of vitiligo [49]. Some studies indicated a positive correlation between the increased number of Th17 cells and the level of IL-21 and TGF-β1 and the area of the body occupated by depigmented skin patches [50]. It was also showed that the transcription factor for aryl hydrocarbon receptor (AhR) and retinoid-related orphan receptor (RORC), which are involved in the production of IL-22 by cells Th17 and Th22, increased considerably [46,51,52]. The research conducted by Zhen et al. demonstrated enhanced Th1 and Th17 response in the circulatory system of vitiligo patients [45]. In addition, Das et al. confirmed an increased concentration of IL-2, which is a cytokine produced by cells Th1 [53].

Regulatory T cells (Tregs) represent a subset of peripheral CD4^+^T cells responsible for, among others, inhibiting the development of autoimmune diseases. At the same time, they do not influence generalized immunodeficiency. Accumulating studies demonstrate that dysfunctions and/or deficiency of Tregs cells have an impact on occurrence of autoimmune diseases [54]. The analysis of the examination results, obtained by means of flow cytometry, showed a slightly decreased number of these cells in progressive vitiligo patients in comparison with stable patients [55]. This confirms reports on the possible recruitment of the circulating Tregs from the peripheral blood to inflamed skin areas. Other research reported a significant decrease in the amount of Tregs cells in marginal skin lesions in both active and stable forms of disease [56]. Despite this, regulatory T cells in vitiligo patients do not have a sufficient inhibitory effect on proliferation and the cytolytic activity of lymphocytes T CD8^+^. It shows a functional defect of peripheral regulatory T cells in vitiligo patients. These observations suggest that the impairment of Treg cell functions combined with the hyperactivity of lymphocytes T CD8^+^ may be significant for the development of the depigmented skin disorder in question [54,55].

## 4. Tissue Resident Memory Cells (T_RM_)

Tissue-resident memory T cells are a heterogeneous group that was first described about 10 years ago, due to the discovery of T cells which do not demonstrate the capacity to recirculate [57]. This long-living subset include CD8^+^ and CD4^+^ cells, which are phenotypically, functionally, and transcriptionally different from circulating effector memory T cells [18]. It has been proved that the skin of a healthy adult person consists of over 20 billion T_RM_ cells [58,59]. Traditionally, T cells are divided into two major subsets: naïve and memory T cells—this is connected with pathogen exposure. As a result of being stimulated by presented antigen, naïve T cells are transformed into several subsets, including central memory T cells (T_CM_), effector memory T cells (T_EM_) and the recently discovered tissue-resident memory T cells (T_RM_). T_CM_ cells move within lymphoid tissue, while T_EM_ cells circulate between peripheral tissues. T_RM_, in turn, are cells that do not have the ability to recirculate between the vascular system and the tissues in which they reside [59,60]. It is expected that the resident subpopulation of memory T cells can develop in the skin from cells that previously expressed KLRG1 protein [61]. Their primary role is to provide antiviral and antibacterial protection in non-lymphoid tissues [18,62,63,64]. 

The CD8^+^ T_RM_ cells formation process is triggered by IL-7, TGF-β and IL-15 [62,65], wherein IL-15 has proven additional influence on their maintenance [66]. Their main feature is a capacity to survive and stay activated in the lesional skin for at least half a year after infection [67]. Due to the fact that cell residence requires specific environmental conditions, including limited access to oxygen and glucose, T_RM_ cells had to develop a different transcriptional profile, compared to their precursors [5]. Adaptations of this long-living T cells subset among others include the following transcription factors: Hobit, Blimp1, and Runx3. It has been demonstrated that Blimp1 is responsible for regulating cytotoxic capacity by stimulating granzyme B expression, while Hobit is indispensable in the long-term maintenance of this enzyme [68]. IL-15 and the transcription factor T-bet stimulate expression of Hobit factor, whereas Blimp1 is induced by Runx3 [69,70]. Although the role of Runx3 in CD8^+^ T cells is not fully determined, it is suggested that this factor has an impact on the presence of CD69 and CD103 surface markers, so it is essential for T_RM_ cell maintenance [71].

T_RM_ cell population is characterized by specific markers of residency, such as CD49a, CD69 and CD103 (Table 2), though not all T_RM_ cells express these particles [57,72]. Furthermore, the expression of adhesion molecules by CD4^+^ tissue-resident lymphocytes seems to be debatable. Some studies indicate the possibility of expression CD103 and CD69 by resided in the skin CD4^+^ effector T cells after antigen exposure [73,74]. Recent data presented by Wilk et al. demonstrated that infection triggered by *B. pertussis* induces CD69^+^CD4^+^ T_RM_ cells with ability to express CD103 marker during and after infection [73]. Currently, the best described are CD8^+^ T cells with CD69 and CD103 surface markers [62]. CD103 (α-subunit of the α_E_β_7_ integrin receptor) is involved in binding E-cadherin synthesized on epithelial cells and in this way promotes T_RM_ cell retention. In turn, work published by Mackay et al. indicated that CD69 is needed for T_RM_ cell development in nonlymphoid tissues, including skin [75]. Expression of this molecule seems to be stimulated by various factors such as antigens and type I interferon exposure [75]. In addition, CD69 has been found to suppress the function of sphingosine 1-phosphate receptor 1 (S1PR1), which appears to be crucial for T_RM_ cell retention in tissues [75]. CD49a, also known as a very late antigen 1 (VLA-1), is an α-subunit of the α1β1 integrin receptor responsible for the induction of granzyme B, perforin, and IFN-γ expression due to the stimulation of IL-15. Therefore, it determines the high cytotoxic capacities of T_RM_ cells [63]. Importantly, the expression of these molecules can vary between cells, depending on what tissue they are in [61]. T_RM_ cell localization is probably related to the presence of β growth factor (TGF-β), which plays the main role in the expression of CD103 marker and in most cases is essential for tissue-resident lymphocytes development [61,62,63].

T_RM_ cells are formed as a result of stimulating naïve T cells by antigen-dependent signals. The long-living population of T cells in each tissue is able to recognize specific pathogens [76]. Recent studies have shown that T_RM_ cells, after renewed contact with the antigen, begin to divide, thus leading to the formation of new subsets that may be present in the tissues for several months to several years after the stimulus factor disappears. For example, infection-induced CD8^+^ T_RM_ are found in the nasal cavity epithelium up to 3 months after the onset of a respiratory tract infection [77], while in skin attacked by HSV for up to a year [78]. On the other hand, it has been proved that mice kept in pathogen-free and clean barrier facilities had few skin T_RM_ cells [79]. It is generally agreed that T_RM_ cells have the ability to slowly migrate in their environment. Therefore, antigen-specific cells, demonstrating uninterrupted movement in the epidermis, increase the protection of the tissue in which they reside [80]. Due to this property, T_RM_ cells are able to identify a cell with expression of the proper antigen within a few hours [81,82]. In this way, it is possible to distinguish HSV-specific T_RM_ cells present in the skin, rotavirus-stimulated T_RM_ cells in the gut, or specifically for influenza virus tissue-resident lymphocytes localized near the airways and bronchovascular bundles in the lungs [73,83,84]. Furthermore, T_RM_ CD4^+^ have a protective effect on the mucosa of the small intestine, and female genitals. They demonstrate an ability to influence on the course of skin infections involving *Leishmania* or *Candida albicans* by immune response based on IL-17 production [60,73,85]. 

On the other hand, their ability to multiply rapidly due to the antigenic stimulation shows the harmful effects of the influence of T_RM_ cells on the body’s functioning. Furthermore, tissue resident memory T cells can increase their number during an ongoing inflammatory process, even without the presence of pathogens [59]. Considering the facts above, in recent years, studies have found that autoreactive T_RM_ cells may play a role in the pathogenesis of many autoimmune disorders, such as psoriasis, rheumatoid arthritis, allergic contact dermatitis, or autoimmune hepatitis [3,85,86,87,88,89]. Some experiments conducted on mouse models demonstrating the graft-vs.-host disease (GVHD) indicated the presence of T_RM_ cells in the digestive tract [90]. Recent research has been focused on broadening our understanding of the role of T_RM_ cells in the pathogenesis of vitiligo.

## 5. T_RM_ Cells and Vitiligo

A histological analysis of vitiligo patients’ skin biopsy revealed that the margin zones of depigmented lesions were strongly dominated by CD4^+^ and CD8^+^ T cells [91]. The hypothesis that CD8^+^ are critical for melanocyte depletion was formulated after discovering that these cytotoxic T cells isolated from vitiligo patches were able to induce depigmentation in an autologous healthy skin explants ex vivo [92], while the potential role of CD4^+^ cells is still not fully determined [93]. The mechanism by which perilesional CD8^+^ T cells cause melanocytes apoptosis is a consequence of their production of proinflammatory cytokines, especially IFN-γ [92]. Therefore, it was suggested that there was a correlation between CD8^+^ T cells response and the development of vitiligo [92]. In addition, patients with stronger CD8^+^ activity required a longer treatment before the first results were visible. This observation implies a connection between the reactivity of melanocyte antigen-specific T cells in the skin and the appearance of depigmented areas [92]. Interestingly, many of the CD8^+^ T cells presented within vitiligo patches possessed a T_RM_ cell phenotype.

The role T_RM_ cells play in the pathogenesis of vitiligo is proven by the fact that skin lesions often appear in the same places they occurred before treatment. A similar phenomenon is observed in psoriasis [85]. It was suspected that T_RM_ CD8^+^ may take active part in blocking repigmentation, by inhibiting melanin-producing cells in the epithelium. Additionally, they probably affect the regeneration of melanocytes by blocking local regulatory T cells (Tregs), which play a vital role for hair follicles stem cells in this process [94]. Latest findings confirm the existence of autoreactive CD8^+^ cells with CD103^+^CD69^+^CD49a^+^ T_RM_ phenotype in the skin of vitiligo patients [4,28,40,84]. Furthermore, melanocyte-specific CD8^+^ T_RM_ cells were highly present in lesional skin as compared to blood regardless of whether the actual disease status was active or stable [86]. 

**Figure 1:**
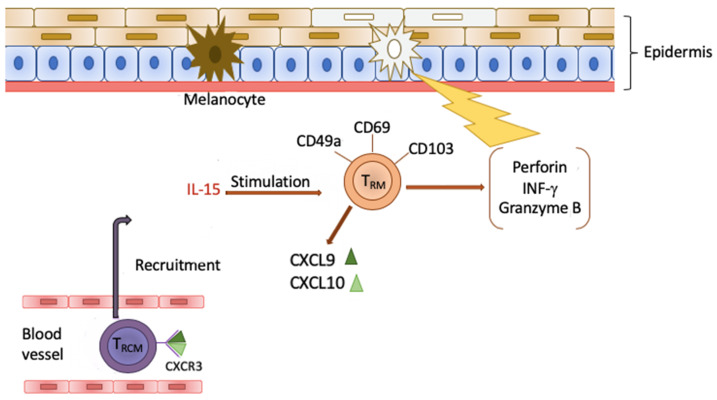
T_RM_ cells demonstrate expression of CD49a, CD69 and CD103 particles. Due to the stimulation of IL-15, they secreting perforin, granzyme B and IFN-γ, indicating in this way a cytotoxic effect on melanocytes. Additionally, T_RM_ produce chemokines CXCL9 and CXCL10, which, after binding with receptor CXCR3 on the surface of T_RCM_ cells, influence their recruitment from the blood vessels to the skin. Next, through the synergistic effect, they cause the depigmentation of the skin in the course of disease.

Research from 2013 [62] focused on receptor CXCR3 (chemokine receptor CXCL9 and CXCL10), which is important for the location of the effector T cells in the epithelium, as well as for the growth of T_RM_ cells. Due to the affinity of CXCL9 and CXCL10, chemokines for receptor CXCR3 found on the surface of T_RCM_ cells (recirculating T_RM_) are able to recruit them to the skin. Next, T_RM_ and T_RCM_, through the synergistic effect and secretory properties of IFN-γ, trigger depigmentation of the affected skin patches on the body. It is interesting that the circulating CXCR3^+^ CD8^+^ T_RCM_ cells in vitiligo patients show increased proliferation potential compared to the cells in healthy people [86]. Moreover, it was observed that the inhibition of T_RCM_ recruitment to the skin had reversed the symptoms of the disease [95]. Considering the role of the chemokines and their receptor, the attempts to base the therapy on targeting the CXCL9/10-CXCR3 path may potentially prove very effective in vitiligo treatment. In addition, some studies revealed the presence of CXCR3 on the majority of CD8^+^ T_RM_, including the cells showing specificity for melanocyte receptors and antigens. Those cells had secretory properties IFN-γ and TNF-α [86]. Other discoveries indicated that IFN-γ and granzyme B are the key cytokines partaking in the course of vitiligo, because they stimulate the apoptosis of melanocytes [96]. These findings show the significant role of T_RM_ cells in the pathogenesis of vitiligo (Figure 1), which explains the growing interest in them in the quest for new treatments.

## 6. Clinical Significance of T_RM_ Cells

Some studies have demonstrated a correlation between T_RM_ cells and IL-15. It was shown that the formation of T_RM_ largely depends on IL-15, and IL-15 promotes the function of T_RM_ cells ex vivo [66]. Moreover, continuous signaling of this cytokine is indispensable for skin T_RM_ cells to survive. Thus, in the course of developing new therapeutic methods, subsequent researcher were focused on this interleukin. Three forms of IL-15 receptor can be distinguished: a monomer, heterodimer, and heterotrimer. The heterotrimeric IL-15 receptor consists of CD122, CD215, and CD132 subunits. This receptor is mainly expressed on NK cells, whereas the heterodimeric receptor is composed of CD122 and CD132 molecules. It is generally present on memory T cells population. What is more, both human and mouse T_RM_ cells which exist in skin lesions present elements of the IL-15 receptor [66]. Furthermore, specific for melanocytes, T_RM_ cells demonstrate expression of CD122. It was found that using CD122 antibody reversed the disease in mice with vitiligo [62,65,66,69]. A 2-week short-term treatment decreased IFN-γ expression, while an 8-week long-term blockade of the IL-15 receptor caused the reduction of autoreactive T_RM_ cells [66]. This observation proves that targeting the IL-15 signal transmission may be an effective method of treatment, both in vitiligo and, perhaps, other diseases where T_RM_ cells play a role in a pathogenesis.

Another trend shows that the life functions of T_RM_ cells also depend on exogenous lipids uptake, as well as their oxidative metabolism. CD8^+^ T_RM_ demonstrate overexpression of lipoprotein lipase (lpl), CD36 (a lipid scavenger receptor) and fatty acid-binding proteins (FABP4 and FABP5), which are molecules facilitating exogenous FFAs acquisition and metabolism [84]. Interestingly, such activity of these molecules is not present in naïve T cells or effector memory T cells [84]. It is proved that cells in vivo with a disability of uptaking exogenous FFAs died prematurely. Additionally, it was indicated that administering medication blocking lipid metabolism decreases T_RM_ cells survivality. For example, etomoxir treatment inhibits the activity of CPT-1 (carnitine palmitoyltransferase-1), which is an enzyme on the mitochondrial membrane. As a result, fatty acid import into these organelles is reduced. Summarizing, CD8^+^ T_RM_ cells which are unable to metabolize exogenous FFAs because of deficiency of FABP4 (Adipocyte—FABP), FABP5 (Epiderma—FABP), or blocking mitochondrial β-oxidation cannot remain in the skin. The lack of fatty acid-binding proteins affects long-term survival of CD8^+^ T_RM_ cells in the skin but does not have any influence on cytotoxic T cells proliferation or recruitment [97]. These observations suggest that by interfering with lipid metabolism processes, it may be possible to manipulate the number of tissue-resident lymphocytes from peripheral tissues during treatment of chronic inflammatory conditions [84,97].

## 7. Anti-Tumor Role of T_RM_ Cells

T_RM_ cells demonstrate an ability to stimulate protective immune responses against pathogen reinfection. They represent a major line of defense by recruiting memory T cells and B cells to the site of ongoing inflammation [67,82,98]. The latest studies demonstrate another very promising function of T_RM_ cells in antitumor immunity. Nevertheless, this phenomenon is not fully understood yet. Some studies with a mouse model shed some light on tumor-specific CD8^+^ T_RM_ cells which might have an inhibitor effect on highly aggressive melanoma. Tissue-resident memory T cells, driven by a model of autoimmune vitiligo, were capable of slowing down the growth of melanoma, in a CD103-dependent manner [99]. In addition, multiple groups’ observations indicated that infiltration of CD8^+^ T cells with a T_RM_ phenotype has an impact on patients’ increased survival in endometrial, breast and high-grade serous ovarian cancer (HGSC) [100,101,102]. Gálvez-Cancino et al. demonstrated that intradermal vaccination is able to stimulate generation of skin-T_RM_ cells from their memory precursors. This vaccination-provoked long-living subset was responsible for significant suppression of B16F10 melanoma [99]. Similarly, OVA-expressing melanoma progression was delayed by injecting recombinant vaccine virus, expressing full-length ovalbumin (OVA) protein. As a result, CD8^+^ T_RM_ and recirculating memory T cells (T_CIRC_) were generated. Intraperitoneal vaccination demonstrated that both T_CIRC_ and T_RM_ cells were able to protect against B16-OVA re-development [103].

## 8. Conclusions

Discovering the T_RM_ cells and connecting their occurrence to the pathogenesis of autoimmune skin diseases allowed the researchers to understand the complicated relations between melanocytes and keratinocytes. Due to recent studies focusing on tissue-resident memory T cells, we will hopefully be able to apply new effective therapeutic strategies in vitiligo treatment in the near future. Identifying the T_RM_ cells precursors and describing their mechanisms helped to broaden the knowledge about their influence on certain processes in the human body, which seems to be a very promising trend.

Though the specific contribution of T_RM_ cells is not unreservedly recognized, emergency reports point to the growing significance of these cells in the development of cancer diseases. It seems that manipulation of tissue-resident lymphocytes might be a promising target for future anti-tumor therapies.

## Figures and Tables

**Table 1:** Vitiligo classification (adapted from Ezzedine et al. [20] based on Bordeaux VGICC * classification and consensus nomenclature).

Type	Subtypes
Non-segmented vitiligo/vitiligo	Acrofacial Generalized Universal Mucosal (more than one site) Mixed (coexistence of SV and NSV) Rare forms
Segmented vitiligo	Unisegmental Bisegmental Plurisegmental
Undetermined/unclassified vitiligo	Mucosal (one site) Focal

* Vitiligo Global Issues Consensus Conference.

**Table 2:** Surface markers of T_RM_ cells and their main functions.

Function			
	CD4^+^/ CD8^+^	Co-receptors of the T cell receptor (TCR) [59]	
**Surface marker**	CD 49a^+^		Secretion of granzyme B, perforin, IFN-γ and achieving high cytotoxic properties following IL-15 stimulation [63]
	CD 69^+^		Blockade of the sphingosine 1-phosphate receptor 1 (S1PR1) [75]
	CD 103^+^		Participation in binding TRM cells to E-cadherin to promote retention within epithelial tissues [72]
